# Male Predominance in West Virginia Unintentional Overdose Deaths Is Influenced by Alcohol and Co-Intoxicants

**DOI:** 10.15288/jsad.24-00054

**Published:** 2025-05-06

**Authors:** Zheng Dai, Marie A. Abate, Mohammad A. Al-Mamun, James C. Kraner, Allen R. Mock, Gordon S. Smith

**Affiliations:** ^a^ Health Affairs Institute, West Virginia University, Morgantown, West Virginia; ^b^ School of Pharmacy, West Virginia University, Morgantown, West Virginia; ^c^ West Virginia Office of the Chief Medical Examiner, West Virginia University, Charleston, West Virginia; ^d^ School of Public Health, West Virginia University, Morgantown, West Virginia

## Abstract

**Objective:**

The purpose of this study was to examine sex differences in overdose (OD) mortality based on substances involved.

**Method:**

We conducted a retrospective database analysis of West Virginia OD decedents (12,666 unintentional OD deaths, 2005–early 2023). Exposures were substances judged to contribute to death. The main outcome measure was determination of male to female death ratios with varying co-intoxicant involvement, particularly related to alcohol and fentanyl. Secondary outcomes included associations of fentanyl concentrations with alcohol concentrations and male sex, including fentanyl and inactive metabolite norfentanyl concentration variability between sexes.

**Results:**

Alcohol co-intoxication in OD deaths was associated with higher male:female death ratios, from 2.0 (alcohol absent) to 3.3 (alcohol present). There was a greater increase over time in alcohol involvement in recent deaths involving females compared with males (relative increases of 52% vs. 6%, respectively). Male:female ratios with alcohol and fentanyl co-involvement ranged from 5.9:1 (only two drugs involved) to 2.4:1 (≥5 substances), with females significantly more likely to have multiple substances contributing to death. Overall, males had statistically significantly larger fentanyl to norfentanyl median concentration ratios compared with females (8.8 vs. 6.9, respectively). Multivariable analyses found that alcohol presence was associated with a statistically significant 22% reduction in predicted fentanyl concentrations.

**Conclusions:**

Male:female ratios in unintentional OD deaths were higher with greater alcohol involvement and lower with fewer co-intoxicants. Fentanyl and norfentanyl concentration differences by sex were observed. It is important to determine possible contributors to sex differences in OD death rates to better target prevention and treatment initiatives.

More than 106,000 drug overdose (OD) deaths occurred in the United States in 2021, with higher rates in men compared with women (Han et al., 2022). Higher drug OD rates in men have also been reported in other countries. The 2023 European Drug Report identified an overall male:female ratio for drug-induced deaths of 3.8 (i.e., deaths occurring in 79% of males vs. 21% of females; European Monitoring Centre for Drugs and Drug Addiction [EMCDDA], 2023). The Australian National Drug & Alcohol Research Centre in 2020 found a lower male:female ratio for overdose and other drug-induced deaths compared with Europe, but their reported ratio of 1.8 (64% vs. 36% deaths in males and females, respectively) still indicated an almost twofold higher death rate in men (Chrzanowska et al., 2022). Based on a recent analysis by Butelman et al. (2023) of U.S. OD mortality from 2020 to 2021 and survey data on drug use, the sex disparity does not appear to result from differences in drug use. The OD fatality rates for men were found to be two to three times higher than for women even after controlling for age and prevalence of opioid and stimulant misuse. It was suggested that behavioral, sociological, biological, or other factors could contribute to higher OD mortality risks among male drug users. Limitations of this study were analysis of only single drug involvement on death certificates, inclusion of suicides (in which drug involvement is generally more common in females), lack of consideration of differences due to multiple drug combinations, not including substance concentrations, and no analysis of the potential contribution of alcohol involvement. In West Virginia, the population at risk for a fatal OD appears fairly balanced between males and females as reflected by a male:female population ratio of 0.98 (Neilsberg Research, 2024) and 1.2 for recent OD-related emergency room (ER) visits (West Virginia Office of Drug Control Policy [WVODCP], 2024). Survey data from 2021 also found a male:female ratio of 1.1 for prevalence of any substance use in West Virginians during the prior 12 months (Mountain State Assessment of Trends in Community Health [MATCH], 2023).

Opioids are frequently identified in unintentional OD deaths. Synthetic opioids other than methadone, involving primarily fentanyl, fentanyl analogs, and tramadol, accounted for the largest increase in opioid-related deaths in the United States from 2016 to 2022 (Spencer et al., 2024). Polysubstance use is also a well-known risk factor for OD fatality because of additive or multiplicative interactions (Compton et al., 2021). However, sex differences in polysubstance use are unclear, although adult men in general were found to be more likely to report polysubstance use (Goodwin et al., 2022).

In addition to numbers of drugs involved in deaths, little is known about whether there are sex differences with various substance combinations, such as those involving alcohol. Opioids and alcohol can each cause fatal respiratory depression, but alcohol ingested with opioids increases the risk of opioid-induced respiratory depression (Bohnert et al., 2011; Jones et al., 2014). An analysis of death certificate data found that alcohol involvement in fentanyl OD deaths increased nearly 60% in 2020 compared with 2019 (White et al., 2022). A limitation of this report is that death certificate analyses likely underestimate the contributing substances in OD deaths because of variability in death certification systems among U.S. states, with no reporting of individual substances involved in ODs on about 10%–20% of death certificates (Hedegaard et al., 2018; Jones et al., 2018; Slavova et al., 2015; Warner et al., 2013). Since an analysis of more than 20 years of U.S. alcohol mortality data found an overall male:female ratio of 2.88 for alcohol-attributed deaths (Karaye et al., 2023), alcohol might be an under-recognized contributor to identified sex differences in fatal overdoses.

Although not fully explored, sex differences in drug clearance and metabolism might affect the concentrations of substances found in decedents (Butelman et al., 2023; Goodwin et al., 2022; Greenblatt & von Moltke, 2008; Ho, 2020; Mazure & Fiellin, 2018). Fentanyl is metabolized rapidly, predominantly by cytochrome P450 3A4 to its major metabolite, inactive norfentanyl (Labroo et al., 1997). Preliminary studies have found increased activity and expression of the P450 3A4 enzyme in females (Hunt et al., 1992; Zanger & Schwab, 2013), with possible increases in the clearance of some P450 3A4 substrates (Cotreau et al., 2005). Whether fentanyl concentrations vary in male and female decedents has not been examined. Thus, the aim of this study was to use a statewide medical examiner system and a comprehensive toxicology database to explore sex differences in unintentional OD mortality.

## Method

West Virginia uses a centralized medical examiner system, and the West Virginia Office of the Chief Medical Examiner (WV OCME) maintains files for all West Virginia deaths. The Forensic Research Data (FRD) began in 2005 in collaboration with the WV OCME to compile data from all West Virginia drug deaths including ODs and transportation or other injury-related deaths for which a drug/substance was considered by the medical examiner to cause or contribute to death. The FRD content has been previously described (Dai et al., 2020). Drug-related death data are entered into the FRD as decedent files are closed; data are currently available from 2005 through early 2023. From the total FRD drug-related deaths, transportation or other injury-related deaths were excluded since the involved substance concentrations might be lower and not the underlying cause of death, e.g., the concentrations might not have resulted in fatality in the absence of trauma. Suicides or ODs with an undetermined manner of death were also excluded since substances were consumed intentionally to cause harm and concentrations would likely be skewed (i.e., higher) and not reflective of an accidental OD. Thus, all unintentional OD deaths constituted the study sample.

Sources used for the FRD data include the death certificate, autopsy reports, external examination and investigator reports, medical records where available, police reports, toxicology reports, and prescription data from the West Virginia controlled substances monitoring program or other information in the decedent's file for noncontrolled substances. Medical history information includes comorbid conditions, diseases, and significant autopsy findings (e.g., atherosclerosis, hepatic necrosis, cirrhosis, etc.) from the sources listed above.

Toxicological testing is routinely performed on all deaths handled by the WV OCME with confirmative toxicology tests conducted for most positive screens, including therapeutic and nonprescription drugs. Blood and/or tissue from decedents are screened for volatile compounds such as ethanol using gas chromatography with flame ionization detection and drugs of abuse using an automated enzyme immunoassay. This latter test includes the following drugs/drug classes: amphetamines, barbiturates, benzodiazepines, buprenorphine, cocaine, fentanyl/fentanyl analogs, other opiates (morphine, codeine, hydrocodone, hydromorphone, oxycodone, oxymorphone), and marijuana. Drugs/drug classes that screen positive undergo confirmation testing and quantitation using liquid chromatography with detection by tandem mass spectrometry (LC/MS). Cases are also screened for therapeutic drugs using LC/MS and/or LC TOF/MS (liquid chromatography time-of-flight mass spectrometry) or LC QTOF/MS (liquid chromatography quadrupole time-of-flight mass spectrometry). Therapeutic drugs screened include the following classes: antihistamines, decongestants, muscle relaxants, bronchodilators, local anesthetics, expectorants, anti-hypertensives, anxiolytics, antipsychotics, antiarrhythmics, antidepressants, anticonvulsants, anti-inflammatories, hypnotics, and antitussives; drugs identified in these screens are also confirmed and quantitated. The term *co-intoxicant* is used to refer to a substance in an OD death when at least one other substance is involved. Fentanyl analogs were tested beginning in 2013, with certain analogs such as acetyl fentanyl readily detected in the immunoassay. Screening for fentanyl (parent) and norfentanyl (inactive metabolite) remained consistent over the study period with quantitation limits of 0.5 ng/ml for both. Femoral or subclavian blood samples were used for all concentration analyses.

The primary outcome was determination of male:female ratios in unintentional OD deaths based on the specific concomitant substances identified, particularly involving alcohol and fentanyl. The secondary outcomes were comparison of fentanyl and norfentanyl concentrations in male and female decedents based on the number of substances present and to examine the association of alcohol concentrations with predicted fentanyl concentrations. The fentanyl to norfentanyl concentration ratio (F:N ratio) was calculated as an approximation of acute versus chronic fentanyl ingestion and to explore sex differences. With acute ingestion and rapid death, higher F:N ratios are expected because of less time for fentanyl metabolism to norfentanyl to occur.

Male:female ratios in deaths involving alcohol were determined by year from 2005 forward to analyze changes over time. Descriptive analyses included the number and types of substances involved in deaths, with male:female ratios determined for alcohol and fentanyl. Wilcoxon rank sum tests compared differences in median F:N ratios between males and females. A multiple linear regression model examined the association of fentanyl concentrations as an outcome with a two-level alcohol concentration cutoff of .08% (w/v), adjusting for demographics (sex, age, body mass index) and toxicological characteristics (presence of multiple opioids, benzodiazepines, stimulants; Dai et al., 2020). The cutoff of .08% was used since it is the U.S. legal limit for driving under the influence. Fentanyl concentration outcomes were log transformed because of skewed distributions. Average percent changes in predicted fentanyl concentrations were calculated based on the corresponding coefficient *b* using the formula (*e^b^* – 1) × 100% to present the findings in a clinically relevant manner. A simple linear regression model was used to assess the association of sex and fentanyl concentrations. We followed the Strengthening the Reporting of Observational Studies in Epidemiology (STROBE) reporting guideline. All analyses were performed using SAS software Version 9.4 (SAS Institute Inc., Cary, NC). Since the study analyzed de-identified decedent data, it is considered non–human subject research by the West Virginia University Institutional Review Board.

## Results

### Number of substances involved

**Figure 1.**
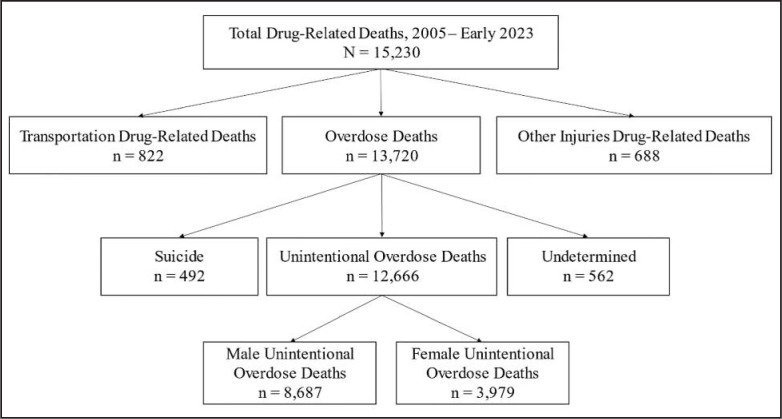
Flow chart of drug-related deaths, 2005–mid 2023. Drug-related deaths from transportation, other injuries, suicide, and undetermined deaths were excluded, resulting in the study population of unintentional overdose deaths (*n* = 12,666).

A total of 12,666 unintentional OD deaths identified in the FRD between 2005 and early 2023 constituted the main study sample (Figure 1). The overall male:female ratio for these deaths was 2.2 (Table 1). The male:female ratios for the excluded drug-related deaths are shown in Supplemental Table A; transportation/injury drug deaths occurred much more frequently in males, and suicide drug deaths involved more females. (Supplemental material appears as an online-only addendum to this article on the journal's website.)

**Table 1.**
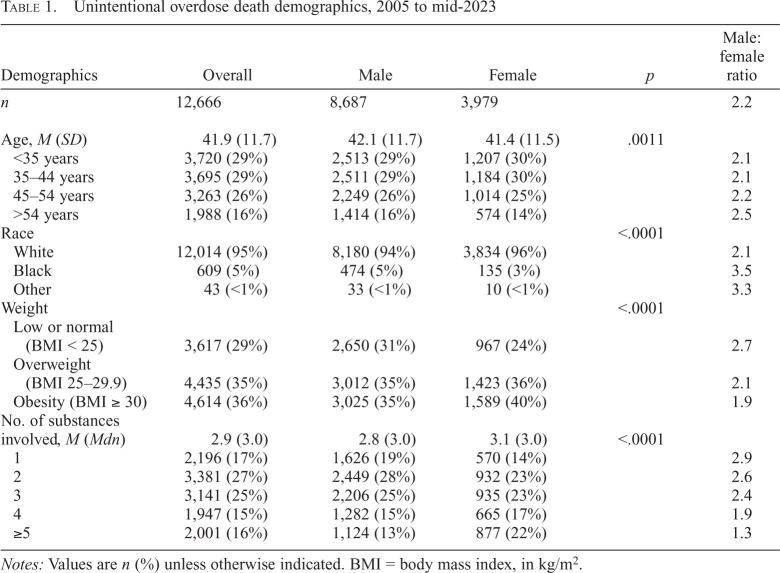
Unintentional overdose death demographics, 2005 to mid-2023

Demographics	Overall	Male	Female	*p*	Male: female ratio
*n*	12,666	8,687	3,979		2.2
Age, *M* (*SD*)	41.9 (11.7)	42.1 (11.7)	41.4 (11.5)	.0011	
<35 years	3,720 (29%)	2,513 (29%)	1,207 (30%)		2.1
35–44 years	3,695 (29%)	2,511 (29%)	1,184 (30%)		2.1
45–54 years	3,263 (26%)	2,249 (26%)	1,014 (25%)		2.2
>54 years	1,988 (16%)	1,414 (16%)	574 (14%)		2.5
Race				<.0001	
White	12,014 (95%)	8,180 (94%)	3,834 (96%)		2.1
Black	609 (5%)	474 (5%)	135 (3%)		3.5
Other	43 (<1%)	33 (<1%)	10 (<1%)		3.3
Weight				<.0001	
Low or normal (BMI < 25)	3,617 (29%)	2,650 (31%)	967 (24%)		2.7
Overweight(BMI 25–29.9)	4,435 (35%)	3,012 (35%)	1,423 (36%)		2.1
Obesity (BMI ≥ 30)	4,614 (36%)	3,025 (35%)	1,589 (40%)		1.9
No. of substances involved, *M* (*Mdn*)	2.9 (3.0)	2.8 (3.0)	3.1 (3.0)	<.0001	
1	2,196 (17%)	1,626 (19%)	570 (14%)		2.9
2	3,381 (27%)	2,449 (28%)	932 (23%)		2.6
3	3,141 (25%)	2,206 (25%)	935 (23%)		2.4
4	1,947 (15%)	1,282 (15%)	665 (17%)		1.9
≥5	2,001 (16%)	1,124 (13%)	877 (22%)		1.3

*Notes:* Values are *n* (%) unless otherwise indicated. BMI = body mass index, in kg/m^2^.

Of the unintentional OD decedents, more than 95% were White; the mean age was 41.9 years, with males slightly older than females (42.1 vs. 41.4, *p* = .0011; Table 1). More than two thirds were either overweight or obese, with a greater proportion of female decedents being obese. The male:female ratios significantly decreased (*p* < .0001) as the number of substances involved in the deaths increased.

**Figure 2.**
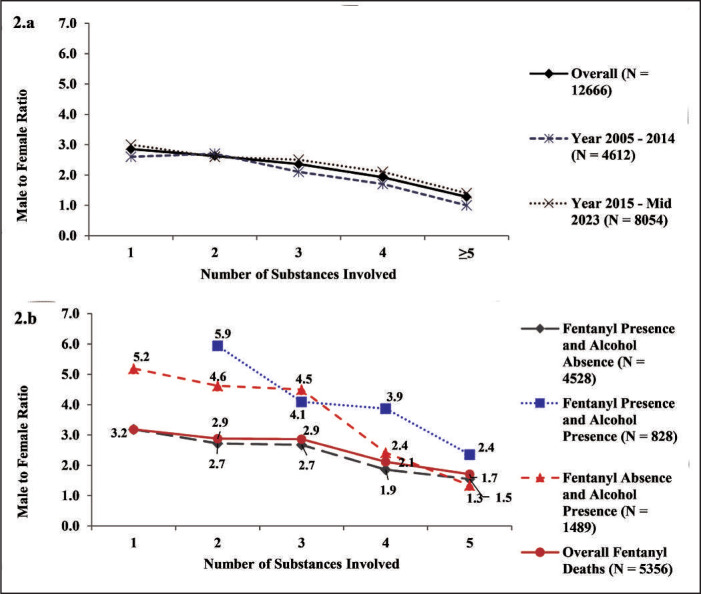
Male:female ratios of unintentional overdose deaths, overall and separated by 2005–2014 and 2015–mid-2023. Figure 2a plots the male:female ratios as the number of substances involved increased for all unintentional OD deaths, overall and by year periods 2005–2014 and 2015 onward. Figure 2b plots the male:female ratios (*y*-axis) as the number of substances involved increased for overall fentanyl deaths, and for fentanyl or alcohol presence or absence.

Figure 2a shows the male:female ratios by year (overall, 2005–2014, 2015 to mid-2023) and number of substances involved. The ratios were slightly higher during more recent years (2015 to mid-2023), but the ratios in both periods were generally parallel as the number of drugs involved increased: one drug (3.0 vs. 2.6), two drugs (2.6 vs. 2.7), three drugs (2.5 vs. 2.1), four drugs (2.1 vs. 1.7), and five or more drugs (1.4 vs. 1.0).

### Alcohol involvement

*Alcohol involvement in unintentional OD deaths*. Alcohol was present in more male than female decedents and was the only drug present in 260 deaths (218 male, 42 female; male:female ratio = 5.2; Supplemental Table C). A total of 2,057 decedents had any alcohol involvement (i.e., alcohol identified with one or more other drugs; male:female ratio = 3.3) and 10,349 decedents (male:female ratio = 2.0) had no alcohol present (Supplemental Table B).

Alcohol presence in unintentional OD deaths also increased over time, from 241 deaths during 2005–2007 to 626 deaths from 2020 to 2022 (Table 2), with consistently greater male involvement. Beginning in 2010, the male:female ratio decreased from 4.2 to 2.8, reflecting gradually increasing alcohol involvement in females. Comparing 2017–2019 to 2020–2022, the proportion of alcohol-related deaths in males increased only 1.0% (6% relative increase) compared with a 5.3% increase (52% relative increase) in females.

**Table 2.**
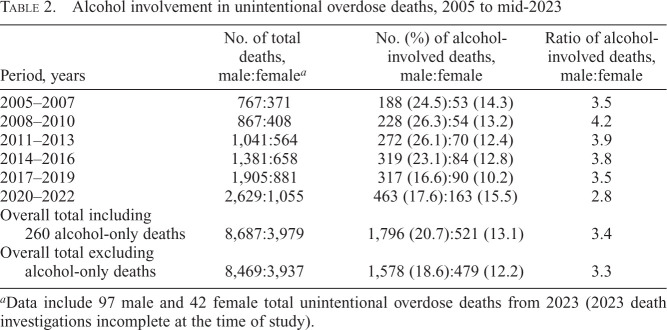
Alcohol involvement in unintentional overdose deaths, 2005 to mid-2023

Period, years	No. of total deaths,male:female*^a^*	No. (%) of alcohol-involved deaths, male:female	Ratio of alcohol-involved deaths, male:female
2005–2007	767:371	188 (24.5):53 (14.3)	3.5
2008–2010	867:408	228 (26.3):54 (13.2)	4.2
2011–2013	1,041:564	272 (26.1):70 (12.4)	3.9
2014–2016	1,381:658	319 (23.1):84 (12.8)	3.8
2017–2019	1,905:881	317 (16.6):90 (10.2)	3.5
2020–2022	2,629:1,055	463 (17.6):163 (15.5)	2.8
Overall total including 260 alcohol-only deaths	8,687:3,979	1,796 (20.7):521 (13.1)	3.4
Overall total excluding alcohol-only deaths	8,469:3,937	1,578 (18.6):479 (12.2)	3.3

^a^ Data include 97 male and 42 female total unintentional overdose deaths from 2023 (2023 death investigations incomplete at the time of study).

Opioids contributed to 85% of unintentional OD deaths, followed by stimulants (40%) and benzodiazepines (35%; Supplemental Table B). With any synthetic opioid or fentanyl involvement, the male:female ratio was 2.5, lower than the proportion of male decedents with alcohol present. There were 55 different two-substance combinations, and 64 three-substance combinations found in the deaths (Supplemental Table C). Alcohol was generally involved in the combinations with the highest proportions of male decedents (Supplemental Table C). Alcohol contributed to death in 56% (*n =* 9) and 42% (*n =* 5) of the 16 two-substance and 12 three-substance combinations, respectively, with a male:female ratio of >5. Among the 10 most frequently involved substances in male and female deaths based on the number of substances present, alcohol was always present in a greater percentage of males than females (Supplemental Table D).

*Alcohol involvement in fentanyl overdose deaths*. Fentanyl was the most frequently identified individual substance in the OD deaths (Supplemental Table D). Figure 2b shows how alcohol presence altered the male:female ratios in fentanyl-related deaths with increasing numbers of substances involved for the following combinations: (a) fentanyl present/alcohol absent, (b) fentanyl and alcohol both present, (c) fentanyl absent/alcohol present, and (d) overall fentanyl-involved deaths. When fentanyl was present without alcohol, the male:female ratios were close to those with fentanyl-related deaths overall. However, when alcohol was present with fentanyl, the male:female ratios were consistently higher (up to almost 6:1) compared to fentanyl without alcohol, even with increasing numbers of other drugs involved.

### Fentanyl concentration analyses

*Ratio of fentanyl and norfentanyl concentrations*. Fentanyl and norfentanyl concentrations were available and analyzed in 84% of unintentional fentanyl-related deaths: 4,497 (3,252 male, 1,245 female) deaths (Table 3). Norfentanyl concentrations were quantifiable in about 84% of female compared with 77% of male decedents. In these cases, males had a slightly lower median fentanyl concentration (0.020 vs. 0.023 µg/ml, *p* < .001) and a significantly lower median norfentanyl metabolite concentration (0.0022 vs. 0.0031 µg/ml) compared with females. This resulted in a significantly higher overall median F:N (parent drug/metabolite) ratio for males than females (8.8 vs. 6.9, respectively). The median F:N ratio difference was greatest when fentanyl was the only substance identified (9.9 vs. 6.7, *p* = .0011). Males had higher F:N ratios than females regardless of the number of substances present; only the difference for four-drug involvement did not reach statistical significance. When norfentanyl was absent or less than the detection limit, median fentanyl concentrations were similar between sexes (about 0.009 vs. 0.010) but were about half those when norfentanyl was present (Table 3). About 2.4 times as many males versus females had an F:N ratio >1 (fentanyl concentrations at or exceeding those of norfentanyl); the numbers of males and females were about equal (ratio = 1.3) when the F:N ratio was <1.

**Table 3.**
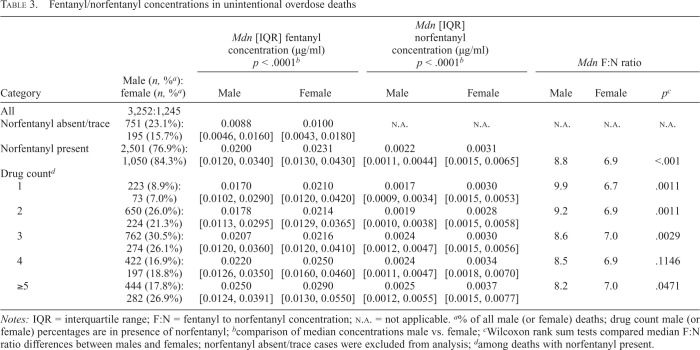
Fentanyl/norfentanyl concentrations in unintentional overdose deaths

Category	Male (*n*, %*^a^*): female (*n*, %*^a^*)	*Mdn* [IQR] fentanyl concentration (µg/ml)*p* < .0001*^b^*		*Mdn* [IQR] norfentanyl concentration (µg/ml) *p* < .0001*^b^*		*Mdn* F:N ratio		
		Male	Female	Male	Female	Male	Female	*p^c^*
All	3,252:1,245							
Norfentanyl absent/trace	751 (23.1%):195 (15.7%)	0.0088 [0.0046, 0.0160]	0.0100 [0.0043, 0.0180]	N.A.	N.A.	N.A.	N.A.	N.A.
Norfentanyl present	2,501 (76.9%): 1,050 (84.3%)	0.0200 [0.0120, 0.0340]	0.0231 [0.0130, 0.0430]	0.0022 [0.0011, 0.0044]	0.0031 [0.0015, 0.0065]	8.8	6.9	<.001
Drug count*^d^*								
1	223 (8.9%):73 (7.0%)	0.0170 [0.0102, 0.0290]	0.0210 [0.0120, 0.0420]	0.0017 [0.0009, 0.0034]	0.0030 [0.0015, 0.0053]	9.9	6.7	.0011
2	650 (26.0%): 224 (21.3%)	0.0178 [0.0113, 0.0295]	0.0214 [0.0129, 0.0365]	0.0019 [0.0010, 0.0038]	0.0028 [0.0015, 0.0058]	9.2	6.9	.0011
3	762 (30.5%): 274 (26.1%)	0.0207 [0.0120, 0.0360]	0.0216 [0.0120, 0.0410]	0.0024 [0.0012, 0.0047]	0.0030 [0.0015, 0.0056]	8.6	7.0	.0029
4	422 (16.9%): 197 (18.8%)	0.0220 [0.0126, 0.0350]	0.0250 [0.0160, 0.0460]	0.0024 [0.0011, 0.0047]	0.0034 [0.0018, 0.0070]	8.5	6.9	.1146
≥5	444 (17.8%): 282 (26.9%)	0.0250 [0.0124, 0.0391]	0.0290 [0.0130, 0.0550]	0.0025 [0.0012, 0.0055]	0.0037 [0.0015, 0.0077]	8.2	7.0	.0471

*Notes:* IQR = interquartile range; F:N = fentanyl to norfentanyl concentration; N.A. = not applicable.

^a^ % of all male (or female) deaths; drug count male (or female) percentages are in presence of norfentanyl;

^b^ comparison of median concentrations male vs. female;

^c^ Wilcoxon rank sum tests compared median F:N ratio differences between males and females; norfentanyl absent/trace cases were excluded from analysis;

^d^ among deaths with norfentanyl present.

*Modeling of fentanyl concentrations*. The multivariable model controlling for demographic and other substance use variables found a statistically significant association between alcohol concentrations ≥.08% (w/v) and a decreased (-22.3%) predicted fentanyl concentration outcome. Simple linear regression found that male sex was associated with a significantly decreased predicted fentanyl concentration (-16.9%) compared with females.

## Discussion

Butelman et al. (2023) provided insight into sex differences seen in U.S. drug OD mortality rates, with reported male:female OD death ratios ranging from 2 to 3:1 depending on the drug involved. Our study found a comparable overall male:female ratio of 2.2 in drug-related deaths. Butelman et al. (2023) further reported that sex differences in OD mortality rates cannot be attributed solely to drug use differences. Using West Virginia data from the MATCH survey (any drug use in past year) and ER visits related to an OD, the population at risk for an OD in West Virginia also appeared similar for males and females (MATCH, 2023; WVODCP, 2024).

This study expanded on prior work examining male:female OD death ratios with additional findings that did not rely on single-cause death certificate data alone: (a) the importance of alcohol in male OD deaths, (b) increasing female involvement (i.e., decreasing male:female ratios) as the number of substances contributing to death increased, and (c) sex differences in fentanyl and norfentanyl concentrations suggesting possible metabolism differences.

Despite its high ranking as a preventable cause of death (National Institute on Alcohol Abuse and Alcoholism, 2024), alcohol is often underreported in drug-related deaths and unrecognized in harm prevention efforts (Lahti et al., 2011; Tori et al., 2020). Male decedents were significantly more likely than females to have alcohol involved in their death. Although opioids were identified in 85% of unintentional OD deaths in our study, alcohol involvement was associated with most of the large sex ratio differences found, with some alcohol–opioid combinations such as alcohol plus buprenorphine, morphine, heroin, or fentanyl present in six or more times as many males as females. Somewhat surprising were the findings of disproportionately larger numbers of males in deaths with combined fentanyl and alcohol or with alcohol involvement without fentanyl compared with fentanyl/no alcohol, although this might be explained to at least some extent by greater alcohol use in general by males.

The finding of increasing alcohol involvement in female decedents over time is consistent with another report (Karaye et al., 2023). Compared with males, physiological differences in females can result in higher blood alcohol concentrations from similar quantities of alcohol ingested, increasing the complication and fatality risk in women. This is concerning given the well-recognized harm potential from combined alcohol with opioids or other central nervous system depressants. A previous study showed that alcohol presence was associated with lower predicted opioid concentrations in combined OD deaths, consistent with a greater risk of harm from smaller concentrations of opioids in the presence of alcohol (Dai et al., 2020).

Unknown adulteration of drugs with xylazine, fentanyl, fentanyl analogs, and other compounds could increase the likelihood of more than one substance being present in OD deaths in addition to knowing drug ingestion (Sibbesen et al., 2023). The literature is conflicting about whether males or females are more likely to engage in polysubstance use. A scoping review found that men engaged more frequently in polysubstance use (Goodwin et al., 2022). In rural populations with opioid use disorder, females were more likely to have multiple opioid prescriptions (Ellis et al., 2021), although a recent review found no substantial evidence that women are more vulnerable to psychostimulant and opioid craving and relapse (Nicolas et al., 2022). Female decedents in our analyses were significantly more likely to have multiple substances (especially four or more; 39% of females vs. 28% of males) involved in OD deaths. As the number of substances involved increased to more than five, the male:female death decreased from 2.2 overall to almost unity. It is unknown whether females are simply more likely to ingest multiple substances in unintentional OD situations or whether interactions might occur among specific drug combinations that could affect females to a greater extent. Men have been documented to engage in riskier types of substance use, including binge drug injection, using substances alone with no others present, combining alcohol with drug use, or repeatedly using contaminated drug paraphernalia (Butelman et al., 2023; Ho, 2020; Minoyan et al., 2023; Norton et al., 2022). Further study is clearly needed to explore potential sex differences in drug-taking behaviors as contributors to OD death disparities.

Males in our study had lower median fentanyl and norfentanyl concentrations than females across the number of substances involved. Relatively lower norfentanyl metabolite concentrations in males resulted in significantly higher fentanyl to norfentanyl concentration male:female ratios, regardless of the number of other substances present. Males were twice as likely to have a F:N ratio >1 (fentanyl concentrations equaled or exceeded norfentanyl concentrations), compared to almost equal numbers of males and females with F:N ratios <1. This was also reflected by the simple linear regression finding of significantly lower fentanyl concentrations in males. One possibility for the concentration differences is that females might metabolize fentanyl more quickly or efficiently to inactive norfentanyl. Increased clearance by females of several P450 3A4 substrates was shown in some human studies; however, the findings and significance were unclear, and fentanyl, also a P450 3A4 substrate, was not studied (Cotreau et al., 2005). Differences in acute versus chronic ingestion might also be a factor. In addition, smaller fentanyl concentrations without detectable norfentanyl might reflect norfentanyl concentrations below the assay detection limit or variability from other sources. Detailed studies of fentanyl metabolism by sex are needed.

A strength of our study is the use of a comprehensive toxicology database from a centralized state medical examiner system that includes data from multiple sources. Other strengths include the availability of more than 18 years of decedent data with detailed information on all substances involved in deaths and the inclusion of primary metabolite concentrations, such as norfentanyl, for drugs involved.

Limitations include the unavailability of fentanyl concentrations for about 16% of cases and the possibility of missing data or entry errors that could occur with any data set, although efforts were made to follow up on missing values or discrepancies to the extent possible. Since it can be difficult to determine suicidal intent, it is also possible that some suicides were mistakenly considered to be unintentional deaths. The study was performed on overdose deaths in one U.S. state, so findings from drugs/drug combinations more commonly found in our deaths might not be generalizable to other regions or countries with different drug use patterns. Illicitly manufactured fentanyl is the substance that caused the greatest number of overdose deaths in the United States in recent years (Spencer et al., 2024), although it is not as prevalent in many other countries (United Nations Office on Drugs and Crime, 2023). For example, heroin was estimated to be involved in almost three quarters of fatal ODs in the European Union in 2023 (EMCDDA, 2023). The extent to which alcohol and multiple drug involvement might similarly influence male:female ratios in OD deaths should be determined in other geographic areas. Finally, causal relationships cannot be established given the observational design of this study.

### Conclusions

Determination of male:female ratios in unintentional OD deaths should account for differences in the number and types of specific substances involved since these affect the ratios reported. Very large male:female ratios were often found when alcohol was involved in the OD deaths. The increasing involvement of alcohol in female decedents in recent years raises the possibility that female death rates will rise in the future. Multiple substance involvement was also found to a proportionally greater extent in female deaths. Variability in fentanyl and norfentanyl concentrations between males and females should be further examined to determine if there are clinically important differences. Recognizing and exploring factors that can contribute to sex differences in OD deaths is essential to tailor OD prevention and treatment initiatives to the unique needs of males and females.

## Conflict-of-Interest Statement

Gordon S. Smith has testified as an expert witness in opioid litigation on behalf of the state of West Virginia.
